# Advancing movement and physical literacy earlier (AMPLE) program for promoting physical literacy and executive function among young children: study protocol of a cluster randomized crossover trial

**DOI:** 10.1186/s12889-025-25753-y

**Published:** 2025-11-26

**Authors:** Zheng Ye, James Robert Rudd, Jia Yi Chow, Cindy Hui Ping Sit, Suzannie Kit Ying Leung, Siu Ming Choi, Minghui Li, Raymond Kim Wai Sum

**Affiliations:** 1 Department of Sports Science and Physical Education, Kwok Sports Building, Chinese University of Hong Kong, Shatin, N.T., Hong Kong S.A.R., China; 2 Department of Teacher Education and Outdoor Studies , Norwegian School of Sport Sciences, Sognsveien 220, Oslo, 0863 Norway; 3 National Institute of Education, Nanyang Technological University, 1 Nanyang Walk, Singapore City, 637616 Singapore; 4 Department of Curriculum and Instruction, Chinese University of Hong Kong, Room 214, Shatin, N.T, Hong Kong S.A.R., China; 5 Faculty of Education, University of Macau, Room 4004, E33, Avenida da Universidade, Taipa, Macau S.A.R., China; 6 Faculty of Physical Education, Ningbo University, No.818 Fenghua Road, Ningbo City, China

**Keywords:** Physical literacy, Executive function, Fundamental movement skills, Cognitive engagement, Physical activity, Holistic development, Preschooler, Early childhood education, Kindergarten, Cluster randomized crossover trial

## Abstract

**Background:**

The positive impact of fundamental movement skills on physical activity in young children has been shown in previous studies, but little is known about the association between fundamental movement skills and cognitive development in young children. This study protocol describes a 10-month, four-arm cluster randomized crossover trial evaluating the effectiveness of the “Advancing Movement and Physical Literacy Earlier” (AMPLE) program in promoting physical literacy and executive function among 3-5-year-old children in 18 Hong Kong kindergartens.

**Methods:**

This program integrates fundamental movement skills training with cognitively challenging activities. Four classes per kindergarten will be randomly assigned to one of four conditions: (1) combined fundamental movement skills and cognitive challenge, (2) sedentary with cognitive challenge, (3) fundamental movement skills alone, or (4) sedentary without cognitive challenges. Each condition involves 30-minute sessions, three times per week, with a six-week washout period between the three-week intervention phases. The research team will use multiple objective measures and self-report measures, covering the elements and domains of the assessment of physical literacy and executive function. Three follow-up tests will be conducted within six months after the end of the intervention to evaluate the long-term impact of the program. Intervention implementation and fidelity will be assessed through focus group interviews with teachers and principals.

**Discussion:**

The “AMPLE” program aims to strengthen the theoretical understanding of motor‒cognitive connections, inform early childhood education practices, and contribute to public health strategies promoting lifelong physical activity. Ultimately, the program seeks to provide evidence-based interventions for fostering holistic child development and establishing healthy lifestyle habits.

**Trial registration:**

The protocol has been registered at Chinese Clinical Trial Registry on August 27, 2025, under identifier ChiCTR2500108295.

**Supplementary Information:**

The online version contains supplementary material available at 10.1186/s12889-025-25753-y.

## Background

Early childhood is the opportune time to promote regular physical activity (PA) engagement and cognitive development, which are crucial for reducing the likelihood of obesity and disease and establishing a foundation for academic achievement [1–4]. As a component of complex movement sequences and sport participation [5, 6], there is increasing evidence that interventions based on Fundamental Movement Skills (FMS) are positively correlated with children’s cognitive function development, especially in terms of memory enhancement [7, 8]. Some perspectives also point out that FMS with cognitive engagement may have better intervention outcomes than single PA/FMS engagement does [9, 10]. One possible mechanism is that FMS allows for the activation of neural networks, and a more aroused neural network state contributes to performance when performing cognitive activities, whereas cognitively integrated FMS tasks activate neural networks for complex cognitive tasks, which allows for higher levels of cognition to be trained [11–13].

However, current research on FMS/PA-based executive function (EF) interventions generally suffers from poor methodological quality, with some studies failing to use validated or reliable assessment methods to examine cognitive functions [8]. Moreover, there have been few interventions or assessments for preschoolers, and some studies have addressed only the relationship between FMS and PA participation in preschoolers without considering possible cognitive effects [14, 15]. There are also some studies whose findings are inconsistent with the hypotheses, and this difference deserves to be explored further [16]. For example, studies have shown no benefit of PA engagement on executive function [17–19] and possibly even negative effects [20]. Possible reasons for these discrepancies include insufficient intensity of the cognitive challenges, fatigue from excessive PA intensity, or a loss of positive emotions [18]. EF develops most rapidly between the ages of 5–8 years, so young children may be more sensitive to these issues, and these possible issues pose a challenge for future research.

As more interventional studies have been conducted, a considerable degree of limitation has emerged from purely quantitative studies. Controlling for the intensity or duration of PA interventions alone, for example, does not seem to lead to a uniform conclusion that not all exercise interventions demonstrate a good ability to improve cognitive functioning [21]. As a result, researchers have gradually turned their attention to qualitative characteristics of how to develop more effective PA intervention models while harmonizing cognitive and motor abilities and why some interventions have a greater ability to improve cognitive function than others do [22]. We should assume that not all PA-related interventions can have a significant positive impact but rather need to consider elements such as the quality of intervention and the measurements used for cognitive development that are critical in influencing intervention outcomes [23].

While PA itself is undeniably important, the emerging concept of physical literacy (PL) offers a more comprehensive and promising approach to cultivating positive movement behaviors and maximizing the benefits of PA across the lifespan [24, 25]. PL emphasizes the development of not only physical competence but also the motivation, confidence, knowledge, and understanding needed to value and take responsibility for engagement in physical activities for life [26]. This broader perspective, which acknowledges the interconnectedness of physical, cognitive, affective, and social domains in shaping an individual’s relationship with movement and activity, is helpful for guiding effective strategies to improve the quality of early intervention for promoting PA [27]. Research has shown that taking specific measures to improve the PL of specific populations is effective, but the mechanism is unclear [28–30], and education programs specifically designed for PL assessment are still lacking [31].

The need for effective intervention is particularly acute in Hong Kong, where studies indicate that a significant majority of preschoolers fail to meet recommended physical activity (PA) guidelines from the World Health Organization, leading to a downward trend in their Fundamental Movement Skills (FMS) [32, 33]. The problem is compounded by the lack of a standardized physical education curriculum in Hong Kong kindergartens, creating a significant gap in our understanding of local children’s FMS and EF levels [34].

In response to these challenges, a growing body of evidence supports the idea that FMS interventions integrating cognitive engagement from the perspective of PL may be more effective than single PA/FMS interventions in terms of physical and cognitive outcomes [8]. Such strategies are capable of significantly enhancing young children’s physical outcomes while simultaneously improving their cognitive development [35, 36]. This study introduces the “Advancing Movement and Physical Literacy Earlier” (AMPLE) program, a cluster randomized crossover trial (CRXO) designed to enhance both PL and EF in Hong Kong preschoolers. AMPLE uses an approach that integrates FMS development with cognitively challenging activities. This integrated approach aims to capitalize on the synergistic relationship between motor and cognitive development, maximizing the potential for holistic child development. By targeting both FMS and EF, the AMPLE program aims to not only increase PL and PA participation and improve physical health but also enhance cognitive function, contributing to long-term population and social well-being. This study provides valuable insights into the effectiveness of this integrated approach and contributes to the development of evidence-based interventions for promoting PL, PA and cognitive function in young children. Specifically, the research questions of this study are as follows: (a) What are the effects of the intervention on children’s physical literacy and executive function compared with usual care? (b) What factors may influence the effectiveness and fidelity of the intervention? We hypothesize the following after the intervention and follow-up:

- H1. The participants in the intervention groups will experience greater increases in cardiorespiratory fitness and FMS than their counterparts in the control group, with the greatest improvement in the combination group.
- H2. The participants in the intervention group will experience greater increases in executive function than their counterparts in the control group, with the greatest improvement in the combination group.
- H3. The participants in the intervention group will experience greater increases in affective competence than their counterparts in the control group, with the greatest improvement in the combination group.
- H4. The participants in the intervention group will experience greater increases in social competence than their counterparts in the control group, with the greatest improvement in the combination group.
- H5. The participants in the intervention group will experience greater increases in PA participation levels than their counterparts in the control group, with the greatest improvement in the combination group.

## Methods

### Study design

This study follows the 2025 Standard Protocol Items: Recommendations for Interventional Trials (SPIRIT) guidelines [37] and will evaluate the effectiveness of the “Advancing Movement and Physical Literacy Earlier” (AMPLE) program, a novel intervention designed to promote physical literacy and physical activity among young children in Hong Kong. The study will employ a four-arm cluster randomized crossover (CRXO) trial design, an approach particularly well-suited for interventions delivered within established settings like kindergartens [38]. The CRXO design offers the advantage of controlling for potential cluster-level confounders and allows each participating cluster to experience all intervention conditions, thereby increasing statistical power [39, 40].

### Randomization

According to the Hong Kong Education Bureau, there are 1,026 kindergartens in Hong Kong in 2022–2023 in 18 districts in 3 regions of Hong Kong Island, Kowloon, and the New Territories [41]. In this study, one kindergarten will be randomly selected from each district, and an email and invitation letter will be sent to the kindergarten principals through a computer-generated randomized sequential list (GraphPad Software, Inc.). After the principals agreed to participate, a randomized sequential list will be generated in the same manner for each kindergarten class, and the four participating classes will be finalized. Then a series of documents related to the intervention, including the theoretical background, the study objectives, the research methodology, and the timeline will be sent to the relevant administrators and teachers of the kindergartens. The parents or guardians of the children participating in the intervention will receive documents including ethics approval from qualified institutions, consent forms, research objectives, timetables, etc., and must sign the consent form before the child can be included as a participant in the study.

### Participants

The study will include typically developing kindergarten children aged 3–5 years. Children aged 6 years will be excluded to avoid attrition due to their transition to primary school. The inclusion criteria are as follows: (1) Participants must be aged between 3 and 5 years (preschoolers). (2) Participants must attend a kindergarten in each of the 18 administrative districts in Hong Kong. (3) Participants must be free of any illness or disability that might interfere with their ability to complete the FMS, physical activity, daily tasks, or cognitive tasks required for the intervention. (4) Prior to enrollment in the intervention, informed consent signed by a parent or guardian must be obtained. The exclusion criteria are as follows: (1) Participants out of the age range of 3–5 years. (2) Participants who cannot attend kindergarten regularly. (3) Participants with known medical conditions or disabilities that would limit their participation in completing the FMS, physical activity, daily tasks, or cognitive tasks required for the study intervention and assessments. (4) Participants who did not provide consent from their parents or guardian.

### Sample size calculations

The sample size is calculated using G*Power version 3.1, which is based on the assumed effect size and dropout rates. In a previous systematic review, the effect size of using FMS alone or cognitive challenges alone as an intervention was medium, whereas the effect size of combined FMS and cognitive challenges ranged from small to large [8]; thus, we assumed that the effect size for the current intervention would be medium (f = 0.3), α = 0.05, and power = 0.80, resulting in a total sample size of at least 82 young children [42]. Additionally, we assumed a participant dropout rate of 20%, resulting in a total sample size of at least 103 young children. This study is a four-arm cluster randomized crossover trial that will involve 18 kindergartens from different districts, and it is estimated that there will be 11 children in each class, so the total number will be 18*4*11 = 792, which meets the minimum requirement of the required sample size.

### Intervention strategy

The AMPLE program comprises four intervention arms:

a Combination Group (FMS with cognitive challenge)
  A series of FMS games with cognitive challenges will be implemented by trained teachers during classroom time. Each game will last 30 min and will be played 3 times a week for a total of 3 weeks. An example of a game with cognitive challenges running as FMS training: “connect 4 real version”, a board with 7 columns and 6 rows, 1.2 m long and 1 m wide, will be placed, with all participants standing behind a taped line 15 m away and a timer set for 30 min. The goal of the participants is to place their 4 game pieces on the large board as quickly as possible and keep the pieces in a straight line (horizontal, vertical, or diagonal lines are all allowed). The game is played in pairs, and once a participant has placed their piece and run back behind the tape line, the next participant is allowed to begin [19]. Throughout the game, all participants will be monitored for heart rate to ensure that their heart rate is maintained within 65% to 85% of their maximum heart rate.
b Cognitive Group (Sedentary with cognitive challenge).
  A series of games with only cognitive challenges will be implemented by teachers during classroom time. Each game will last 30 min and will be played 3 times a week. An example of the game is “connect 4 routine version”, all participants will be seated at a table to ensure a sedentary state, and a timer will be set for 30 min. The goal of the game is to place 4 of their own game pieces on the board and try to connect the pieces in a straight line. The game is played in pairs, and once one participant has placed his or her piece, the other participant will be allowed to place the piece.
c Physical Group (FMS without cognitive challenge).
  A series of games with FMS interventions will be implemented by the teachers during classroom time. Each game will last 30 min and will be played 3 times a week. An example of the game is as follows: a clearly marked chart with rounds will be placed, all participants will stand behind a taped line 15 m away, and a timer will be set for 30 min. Once a participant has run out, they need to mark the number of rounds on the chart as soon as possible and return; once they have returned, the next participant is allowed to start and repeat. Throughout the game, all participants will be monitored for heart rate to ensure that their heart rate remains within 65% to 85% of their maximum heart rate.
d Control Group (Sedentary without cognitive challenge).
  All participants will not engage in any cognitive challenge or FMS interventions but will sit in their own seats while the teacher plays a 30-minute story appropriate for young children. All participants will be informed prior to the start of the program that they will not be asked any questions related to the video or story and ensure a state of minimal cognitive engagement.

### Washout period

In a cluster randomized crossover (CRXO) design, washout periods need to be carefully set to minimize the impact of the effects produced by the previous intervention on the next intervention [43]. The carry-over effects of psychological or behavioral interventions are difficult to measure accurately, and the optimal duration for a washout period has not yet been clearly established. In a study published by a team of Stanford University researchers on the effects of digital physical activity intervention on daily steps, the crossover process of four interventions did not have a washout period [44]. Some previous crossover designs related to PA interventions agreed that it is appropriate to set a washout period equal to the duration of the intervention period [45–47]. In addition, there is a need to consider the impact of young children’s own rapid development of motor skills and cognition in non-intervention situations. Therefore, this study hypothesizes that the carry-over effects of the intervention on young children will be greater than the rest of the lifespan, that the duration of the washout period will be set to be twice the length of the intervention period (3 weeks for the intervention period and 6 weeks for the washout period), and that pre-tests and post-tests will be implemented at each stage of the intervention to ensure the accuracy of the measurements

### Data collection procedures

Data collection will follow a cyclical pattern: 1-week pre-test, 3-week intervention, 1-week post-test, and 6-week washout. This cycle will be repeated three times, followed by a final post-test at week 38 and three follow-up tests at 8-week intervals (weeks 46, 54, and 62). See Table 1 for the detailed schedule. In the CRXO design of this study, from the cohort perspective, each cluster is required to receive all 4 interventions sequentially; from the cross-sectional perspective, each cluster is required to receive 4 different interventions each at the same time; thus, a total of 24 combinations of the intervention sequence are generated (4*3*2). Since the effect of the intervention sequence on the results of the study will not be considered, this study will use GraphPad Software to randomly select the combination of the intervention sequence to be used in the study.

**Table 1:** Schedule of the cluster randomized crossover trial

Schedule of Interventions 2025–2026		
20/10/2025-26/10/2025	1 week	Pre-test 1
27/10–16/11	3 weeks	Intervention 1
17/11–23/11	1 week	Post-test 1
24/11/2025-4/1/2026	6 weeks (Including Public Holiday for Christmas)	Washout period 1
5/1–11/1	1 week	Pre-test 2
12/1–1/2	3 weeks	Intervention 2
2/2–8/2	1 week	Post-test 2
9/2–22/3	6 weeks (Including Public Holiday for Spring Festival)	Washout period 2
23/3–29/3	1 week	Pre-test 3
30/3–19/4	3 weeks	Intervention 3
20/4–26/4	1 week	Post-test 3
27/4–7/6	6 weeks	Washout period 3
8/6–14/6	1 week	Pre-test 4
15/6 − 5/7	3 weeks	Intervention 4
6/7–12/7	1 week	Post-test 4
13/7 − 6/9	8 weeks (13/7–31/8 for washout, 1/9 − 6/9 for test)	Follow-up 1
7/9 − 1/11	8 weeks (7/9–25/10 for washout, 26/10 − 1/11 for test)	Follow-up 2
2/11–24/12	8 weeks (2/11–17/12 for washout, 18/12–24/12 for test)	Follow-up 3

The intervention period starts on October 20, 2025, and ends on July 12, 2026, lasting for 38 weeks (10 months). Kindergartens in Hong Kong have Christmas holidays in late December, traditional Chinese New Year holidays in mid-February, and summer vacations in mid-July. As a result, this study will put relatively long vacation periods in the washout period to ensure that all interventions and assessments could be completed in the kindergarten. In addition, in September, teachers may not be familiar with the newly enrolled children, and young children may not yet fully adapt to kindergarten life. Attrition is considered to appear during this period. Thus, the study will start in late October to ensure the smooth implementation of the intervention.

**Fig. 1:**
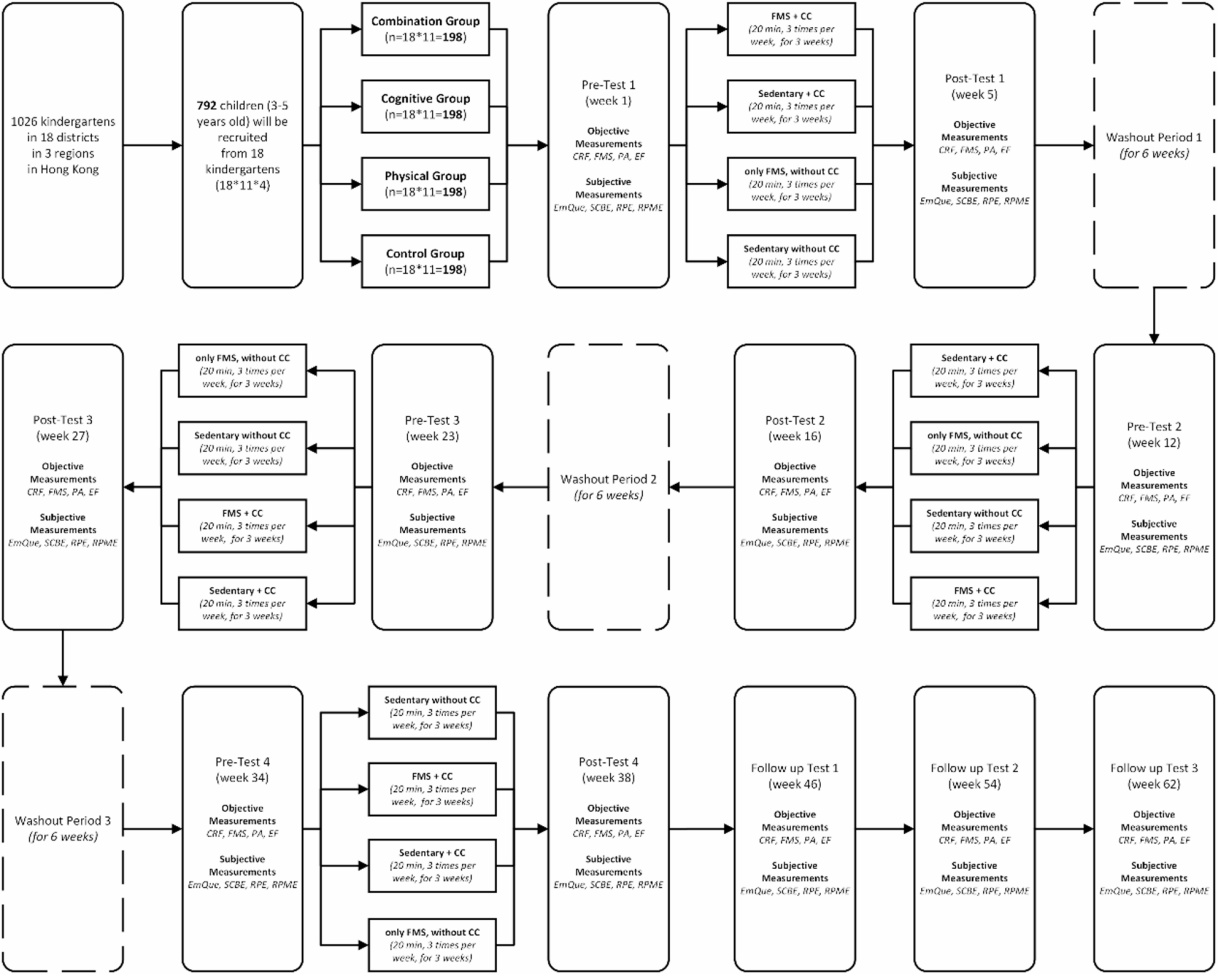
Flow diagram of participants’ progress through different phases. *Abbreviations*. *CC * Cognitive Challenge, *CRF *Cardiorespiratory Fitness, *EF * Executive Function, *EmQue * Empathy Questionnaire, *FMS * Fundamental Movement Skills, *PA * Physical Activity, *RPE * Rating of Perceived Exertion, *RPME * Rating of Perceived Mental Exertion, *SCBE * Social Competence and Behavior Evaluation Scale

Data will be collected through a combination of objective measures and questionnaires and direct observation during the pre-test, post-test and follow-up test periods (see Fig. 1). In addition to professionally trained test evaluators, kindergarten principals and teachers will play an important role in the study. The principal will be the main contact person for the implementation of the study and will be responsible for approving and monitoring the full process of compliance with the program. Teachers in the relevant classrooms will be invited to participate in weekly workshops held by the research team until the end of the intervention. During the workshops, teachers will be carefully instructed on the carefully designed intervention plan for the following week for the different groups, dos and don’t, safety guidelines, etc., as well as how to use, fill out, and keep the evaluation booklet in between interventions. The attendance of all participating teachers will be recorded, which is considered to be one of the important factors that may affect the effectiveness of the intervention. Except for the kindergarten principal, teachers and children will be blinded to the differences in intervention strategies throughout the process, and all assessors will be blinded to group assignment. Unblinding will be permissible after the end of the project.

### Outcome measures

#### Measurement of physically related outcomes

##### Cardiorespiratory fitness (CRF)

The original version of the 20 m shuttle-run test (20mSRT) has been widely used to assess the cardiorespiratory fitness of a cumulative total of more than 1 million children and adolescents worldwide and has been found to be strongly correlated with maximal oxygen uptake [48]. However, its original protocol, which commences at a speed of 8.5 km·h⁻¹, is unsuitable for preschoolers aged 3–5 years, as the high initial intensity can impede performance and compromise test reliability and feasibility. Consequently, some young participants are unable to complete a single shuttle, leading to significant deviations between estimated and actual fitness levels [49].

To address this limitation, this study employs the 20mSRT-PREFIT (20 m shuttle-run test PREschoolers FITness assessment), an adapted protocol developed specifically for this age group [50]. This version modifies the test by reducing the initial speed to 6.5 km·h⁻¹ and incorporates the presence of an adult assessor who runs alongside the children to ensure safety and procedural continuity [51, 52]. CRF has been measured in more than 3,000 preschool children via the 20mSRT-PREFIT, and this protocol demonstrated high reliability (α = 0.833, *p* < 0.01) and greater discrimination compared to the original version in a previous assessment of cardiorespiratory fitness in young children aged 4–6 years [50, 53].

The test requires participants to run from one marked line to another marked line 20 m apart and to run back and forth continuously until exhaustion. They were alerted to this process by an audio signal of progressively increasing speed, set at an initial speed of 6.5 km h^− 1^, which would increase at a rate of 0.5 km h^− 1^ per minute. The test will be stopped when the participant fails twice to reach the marker line at the time of the audio signal or is unable to continue due to fatigue. Later in the test, the participants will be continually encouraged to continue persevering so that their best performance can be recorded. The manual of implementation within video and audio on how to perform and score the 20mSRT-PREFIT is available on the website from the PROFIT Research Group (http://profith.ugr.es/recursos-prefit).

##### Fundamental movement skills (FMS)

The Test of Gross Motor Development Third Edition (TGMD-3) will be used to test participants’ FMS, which is specifically designed for children aged 3–10 years. As a procedure-oriented assessment test tool, it can evaluate the specific performance and completion process of children’s movements, and the operation process is simple and easy to observe. Therefore, it is widely used in the assessment of children’s FMS. TGMD-3 consists of a locomotor skills test and an object control skills test, which together have 13 movements (run, gallop, hop, skip, jump, slide, two-handed strike, one-handed strike, dribble, catch, kick, overhand throw, and underhand throw). The participants will be asked to watch a skill demonstration and be allowed to take a practice test before completing two formal tests. Proficiency the test will be assessed, with 3–5 movement criteria for each move. If a performance criterion for a skill is met, a score of “1” is given; if it is not met, the score is “0”. Each item will be tested twice, and the score will be accumulated for each item. The maximum total original score for the locomotor skill subscale is 46 points, and the maximum score for the object control skill subscale is 54 points. The higher the score is, the better the performance [54]. The assessments will be videotaped and scored by trained professionals [55].

Since the TGMD-3 does not include the assessment of stability skills in young children, the stability skill test (SST) will be used [56]. This test has been used for the assessment of 556 young children and was found to be a reliable scale (α = 0.81) [57]. The SST consists of three parts (rock, log roll and back support), and there are also 3–5 criteria for each move, with each movement tested twice. The final score of the two tests will be added to obtain the final score of the stability skill. The higher the score is, the better the stability skill [56].

#### Measurement of physical activity

PA levels will be assessed via an accelerometer (GT3X+, Actigraph, Florida, USA). This accelerometer is widely utilized in the assessment of PA in children and adolescents and has been shown to be equally effective in young children aged 3–6 years [58]. The participants in this study will be asked to wear the accelerometer for at least 8 h per day for 6 consecutive days, and their PA levels will be categorized as sedentary, light, moderate, or vigorous.

#### Measurement of executive function

##### Working memory (WM)

Working memory will be assessed via the word list recall test (WLR), which is a subset of the children’s working memory test battery and requires participants to memorize and recall word sequences and verbally retell them at specified intervals. As a simple working memory task, participants only need to memorize and repeat the words in the correct pronunciation and sequence without additional manipulation or processing, making it a simple and effective working memory test. Previous assessments using the WLR with preschoolers have demonstrated good retest reliability (α = 0.72–0.8) [59, 60]. In the present study, young children will first hear 2 words that they can understand, and after successfully repeating them with the correct pronunciation and sequence, will add 1 word and repeat the previous maneuver, stopping until two occurrences of an inability to repeat the word or sequence. The number of words that can be recalled accurately will be recorded.

##### Cognitive flexibility & inhibitory control

The Trial Making Test for Preschoolers (TRAILS-P) consists of 4 conditioned tests, with Conditioned Test B being used to assess cognitive flexibility and Conditioned Test C being used to assess inhibitory control. Good reliability (α = 0.64) has been demonstrated in studies with over 100 preschoolers [9, 61]. In Conditional Test B, children will be required to match five dogs and five corresponding bones by stepping on them. In Conditional Test C, the bones serve as distractors, and young children will be required to accurately stamp 10 dogs in order from smallest to largest and to ignore the five interspersed bones. The time taken by the child to complete the task and the number of errors made during the test will be recorded.

#### Measurement of affective/emotional competence

The children’s emotional competence was assessed via the Empathy Questionnaire (EmQue) [62]. The scale was constructed on the basis of the widely accepted Hoffman empathy theory [63] and is divided into three domains: emotion contagion (i.e., being able to pay attention to others’ emotions but not be able to distinguish the subject of the emotion, e.g., My child also needs to be comforted when another child is in pain); attention to others’ feelings (i.e., being able to perceive others’ emotions and distinguish the subject of the emotion, e.g., when an adult becomes angry with another child, my child watches attentively); and prosocial actions (i.e., being able to perceive and distinguish the subject of the emotion and intervene in others, including helping, sharing, and comforting, e.g., when I make clear that I want some peace and quiet, my child tries not to bother me). The 20-item scale uses a 3-point rating scale (0 = never; 1 = sometimes; 2 = often) to assess the child’s behavior over the past two months. It has shown acceptable reliability (α = 0.58–0.81) in previous studies of parents and is significantly predictive of emotion regulation ability [62, 64]. The scale will be completed independently by parents before and after the intervention.

#### Measurement of social competence

The Social Competence and Behavior Evaluation Scale (SCBE-30) will be used to assess the social competence of young children [65]. The scale is divided into three domains: social competence (including joyful, secure, tolerant, socially integrated, calm, prosocial, cooperative, and autonomous); anger aggression (including angry, aggressive, egotistical, and oppositional); and anxiety withdrawal (including depressed, anxious, isolated, and dependent). There are 30 items in total, using a 6-point Likert-style scoring, with 1 representing “never” and 6 representing “always”. Several previous studies have verified that it has good reliability among children in different countries [66–68], especially in assessments of over 1400 Chinese children aged 3–6 years (α = 0.66–0.92) [69, 70]. The scale will be completed independently by teachers and parents before and after the intervention, with the assessment of inter-rater reliability.

#### Measurement of intervention intensity control

The ratings of perceived exertion and perceived mental exertion will be measured with the help of teachers immediately after each intervention to help adjust and identify the intensity of PA and the difficulty of the cognitive challenges that are most appropriate for EF interventions for young children. The Borg Category-Ratio 10 scale (Borg CR-10) [71] has been widely used to assess the intensity of perceived PA, which is a judgment of one’s own bodily effort to complete a given behavior. The scale has 10 levels (1–10), with 1 indicating no exertion and 10 indicating maximum exertion. As the participants are young children aged 3–5 years, the scale will be cartooned, and additional descriptions will be provided to ensure that young children are able to select the most relevant option.

### Evaluation of implementation

A comprehensive implementation and process evaluation will be conducted alongside the CRXO trial. Weekly teacher workshops and fidelity checklists will ensure adherence to the intervention protocols. Session logs and teacher attendance records document the amount of intervention delivered. Focus groups with teachers (6 kindergartens randomly selected, 5–6 people in each group) and interviews with principals (6 kindergartens randomly selected) will explore experiences, challenges, and facilitators related to implementation.

### Data analysis

Descriptive statistics (means and standard deviations) will be calculated for all subscales across all measurements. Missing data will be calculated via maximum likelihood estimation via SAS version 9.4 (Cary, NC, USA). Given the characteristics of behavioral intervention research, we will not assume that the collected data will conform to a normal distribution and will be independent of each other. Therefore, a linear mixed effects (LME) model (from the R library NLME, R version 3.4.4) will be used to analyze the associations of different interventions with EF. The number of intervention days (time) was considered to have a possible association with intervention effects [72], so the interaction between the two will be included in the model. Fixed effects will include the intervention days and group, whereas the cluster (i.e., kindergarten) will be treated as a random effect. The LME model will be generated via the R NLME package. A p value less than 0.05 is recognized as a significant difference. Tukey’s honestly significant difference (HSD) test will be used to determine whether there is a significant difference between the interventions [73]. In addition, the false discovery rate-corrected *p* value will be used to determine if there is a statistically significant difference between the interventions. A value less than 0.05 is considered significant. Secondary outcomes (including cognitive difficulty, PA intensity, and teacher attendance) will be similarly assessed via the LME model.

An exploratory factor analysis of the possible static associations between FMS and EF will be conducted, which will be analyzed via partial least squares structural equation modeling (PLS-SEM), since PLS can maximize the prediction capability [74] and can address formative indicators as well as reflective indicators [75]. The relationship between PL and EF will be assessed via Pearson’s correlation coefficient. The effect size (Cohen’s *d*) will be calculated by dividing the difference between the post-intervention means of the two groups by their pooled standard deviation (SD).

### Timeline

**Fig. 2:**
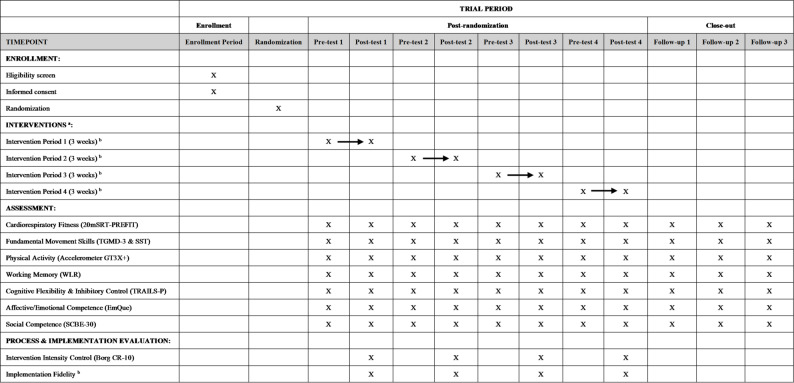
Timeline of enrollment, interventions, and assessments of the “AMPLE” program. ^a ^In each 3-week intervention period, clusters receive one of four conditions: (1) FMS with cognitive challenge, (2) Sedentary with cognitive challenge, (3) FMS without cognitive challenge, or (4) Sedentary without cognitive challenge. As this is a cluster randomized crossover trial, each cluster receives all four conditions sequentially over the course of the study. ^b ^Arrow indicates continuous delivery of intervention.^c^ Implementation Fidelity includes workshops, logs, questionnaires and interviews for teachers.*Abbreviations*: *20mSRT*-*PREFIT* 20 m Shuttle-Run Test PREschoolers FITness Assessment, *Borg CR*-*10 * Borg Category-Ratio 10 Scale, *EmQue* Empathy Questionnaire, *FMS* Fundamental Movement Skills, *SCBE*-*30* Social Competence and Behavior Evaluation Scale, *SST* Stability Skill Test, *TGMD*-*3* Test of Gross Motor Development, Third Edition, *TRAILS*-*P *Trial Making Test for Preschoolers, *WLR * Word List Recall Test

The “AMPLE” program is a large-scale intervention program led by the Department of Sports Science and Physical Education of The Chinese University of Hong Kong, with cooperation from kindergartens in various administrative regions of Hong Kong and guidance and suggestions from domestic and foreign scholars. Figure 2 shows the expected process and timeline of the AMPLE program.

### Patient and public involvement

The design and implementation of this study will be planned and conducted in close collaboration with frontline stakeholders, including kindergarten principals, teachers, and parents, to ensure the ecological validity, relevance, and potential for sustainable implementation of the research. Kindergarten principals and teachers are key partners in this research. They will be involved in preliminary discussions during the development of the study protocol. Their practical experience and pedagogical expertise are invaluable in ensuring that the intervention activities are developmentally appropriate for 3- to 5-year-old children, feasible to implement within a 30-minute class period and can be conducted safely within a standard kindergarten setting. Throughout the intervention period, teachers will participate in weekly workshops where they will not only receive training but also provide continuous feedback on the implementation process. This iterative feedback loop will allow for dynamic and responsive refinements to subsequent activities. Furthermore, post-intervention focus groups with teachers will explore their perceptions of the intervention’s acceptability, as well as the challenges and facilitators encountered. These qualitative data will be crucial for contextualizing the quantitative outcomes and informing future dissemination and scale-up strategies.

Parents are involved in providing key data from questionnaires regarding their child’s emotional and social competence, and they offer a unique perspective on the intervention’s extended impact within the home environment.

This multi-faceted involvement ensures that the “AMPLE” program is not merely an academic exercise but a collaborative study involving schools and homes closely aligned with the local Hong Kong early childhood education ecosystem. It is expected that this approach could significantly enhance the practical relevance of the findings and lay a foundation for the future successful implementation and adoption of the program within the broader early childhood education system.

### Ethics and dissemination

The study protocol has been reviewed and approved by the Joint Chinese University of Hong Kong-New Territories East Cluster Clinical Research Ethics Committee (Ref. No.: 2024.702-T). It has also been registered at the Chinese Clinical Trial Registry (Identifier: ChiCTR2500108295). Prior to data collection, written informed consent will be obtained from the parent/guardian of each participating child. Informed consent will also be obtained from the participating kindergarten principals and teachers. To ensure confidentiality, all participants (children, teachers, and principals) will be assigned a unique code. All data will be anonymized and stored securely, separate from any personally identifying information, on a secure server at the Chinese University of Hong Kong that is compliant with data protection regulations. Data access will be restricted to the principal investigator and authorized research team members. All participants will be informed of their right to withdraw from the study at any time, for any reason, without penalty.

The study findings will be disseminated through peer-reviewed publications, conference presentations, reports to stakeholders (including participating kindergartens and policymakers), and public engagement activities. The “AMPLE” program materials and de-identified dataset will be made publicly available following study completion and primary analyses, respectively, in accordance with the policies of The Chinese University of Hong Kong (CUHK) and the Research Grants Council (RGC) of Hong Kong.

## Discussion

This study protocol details the “Advancing Movement and Physical Literacy Earlier” (AMPLE) program, a four-arm cluster randomized crossover trial designed to effectively improve young children’s physical literacy and cognitive levels, ultimately achieving multiple objectives with a single intervention and laying the foundation for subsequent school learning and lifelong health.

The findings are expected to contribute to theoretical development, particularly concerning the relationship between motor competence and cognitive function. The study is designed to empirically test the foundational theory, rooted in Piaget’s work, that motor and sensorimotor abilities are a precursor to cognitive development [76], which can provide evidence for or against the hypothesis that FMS is an “important starting point of EF” [77]. A key theoretical advancement lies in the potential to elucidate the underlying mechanisms driving this connection. The four-arm design is able to test the “cognitive engagement” hypothesis—the idea that the quality and complexity of physical activity, rather than movement alone, yields the greatest cognitive benefits. The study can directly investigate whether complex FMS tasks activate neural networks for complex cognitive tasks more effectively than FMS training without a cognitive challenge [13]. This could help explain the inconsistent findings in the existing literature, where some studies have reported no cognitive benefits from physical activity [18], possibly due to interventions that lacked sufficient cognitive demands. By clarifying the role of cognitive engagement, this research can help build a more accurate theoretical model of how to optimize interventions for motor-cognitive development among young children.

These theoretical insights can translate directly into implications for the design and implementation of early childhood education curricula. In Hong Kong, In Hong Kong, virtually all children under the age of 5 years enter kindergarten [78]. As a result, the kindergarten environment may be the first choice for implementing intervention programs to achieve specific health goals for young children [36, 79]. Considering the severe challenge of insufficient physical activity faced by young children in Hong Kong, the “AMPLE” program not only helps researchers understand the synergy between FMS and EF but also has high social and educational value. The program’s emphasis on stakeholder collaboration and its implementation within authentic classroom settings ensures that the resulting program materials will be practical and feasible and can better prepare children for the cognitive and social demands of formal schooling. If the combined intervention group demonstrates superior outcomes, it will offer a highly efficient model for educators who must balance numerous learning objectives within a limited timeframe, showing that a single, well-designed set of activities can simultaneously promote multiple domains of health indicators.

From a broader perspective, this research has the capacity to inform public health strategies and policy decisions aimed at promoting lifelong physical activity and addressing the global issue of childhood inactivity [80]. By providing clear evidence of an effective PL-focused intervention, this research can equip policymakers with the justification needed to advocate for the integration of such programs into national early childhood education and public health frameworks. The potential long-term societal benefits are significant. Demonstrating a clear pathway from an FMS-based intervention to improved EF can strengthen the case for investing in early physical education to enhance school readiness and later academic achievement [81]. Furthermore, fostering motor competence and enjoyment of movement in early childhood is critical for establishing a “regular physical activity habit” that can persist throughout life [82]. In summary, the findings from the “AMPLE” program may have a positive impact on improving the quality of early childhood education in Hong Kong, improving public health, and reducing social spending on healthcare, thereby contributing to the development of a healthier and more active future generation.

### Strengths and potential limitations

There are several strengths of this protocol. The four-arm cluster randomized crossover design is particularly suitable for educational research, as it effectively controls for cluster-level confounding variables by allowing each class to serve as its own control. This approach helps manage the influence of cluster-level confounding variables, such as the school environment or teacher characteristics, thereby increasing the statistical efficiency of the analysis [38]. Furthermore, this study addresses the issue of poor methodological quality that has been identified in previous intervention research [8] by employing a comprehensive assessment battery composed of validated and objective measurement tools. The implementation of the program by teachers within their natural kindergarten setting further enhances the ecological validity of the study, making its findings more translatable to everyday educational practice.

There are several foreseeable operational challenges. A principal concern is maintaining intervention fidelity, as it can be challenging to ensure that teachers accurately implement four different intervention programs over a 10-month period. To address this issue, this study included weekly teacher training workshops, a detailed course manual with clear instructions, and the use of questionnaires to monitor compliance. Teachers’ attendance at these workshops will also be recorded and analyzed as a potential factor influencing the effectiveness of the intervention. A 6-week washout period after each intervention also helped reduce teacher fatigue and boredom. Another challenge is the potential for carry-over effects. Although we set a washout period that is twice the intervention period, which is more conservative than previous CRXO studies, there is still no evidence to prove the optimal washout period setting for this type of cognitive or educational research. Finally, as a long-term intervention, there may be a high dropout rate and missing data, as well as a high probability of participant loss at the three follow-up tests after the intervention.

## Supplementary Information

Supplementary Material 1.

## Data Availability

No datasets were generated or analysed during the current study.

## Abbreviations

20mSRT: 20 m Shuttle-Run test
20mSRT-PREFIT: 20 m Shuttle-Run Test PREschoolers FITness Assessment
AMPLE: Advancing movement and physical literacy earlier
Borg CR-10: Borg category-ratio 10 scale
CC: Cognitive challenge
CRF: Cardiorespiratory fitness
CRXO: Cluster randomized crossover trial
EF: Executive function
EmQue: Empathy questionnaire
FMS: Fundamental movement skills
HSD: Honestly significant difference
ICH-GCP: International council for harmonization of technical requirements for registration of pharmaceuticals for human use guideline for good clinical practice
LME: : Linear mixed effects
PA: Physical activity
PL: Physical literacy
PLS-SEM: Partial least squares structural equation modeling
RGC: Research grants council
RPE: Rating of perceived exertion
RPME: Rating of perceived mental exertion
SCBE-30: Social competence and behavior evaluation scale
SD: Standard deviation
SST: Stability skill test
TGMD-3: Test of gross motor development, third edition
TRAILS-P: Trial making test for preschoolers
WHO: World Health Organization
WLR: Word list recall test
WM: Working memory
